# Functional Relevance of Missense Mutations Affecting the N-Terminal Part of Shank3 Found in Autistic Patients

**DOI:** 10.3389/fnmol.2018.00268

**Published:** 2018-08-07

**Authors:** Fatemeh Hassani Nia, Hans-Jürgen Kreienkamp

**Affiliations:** Institute for Human Genetics, University Medical Center Hamburg-Eppendorf, Hamburg, Germany

**Keywords:** ankyrin repeat, intramolecular interaction, dendritic spine, f-actin, fodrin, mGluR5, ras proteins

## Abstract

Genetic defects in *SHANK* genes are associated with autism. Deletions and truncating mutations suggest haploinsufficiency for Shank3 as a major cause of disease which may be analyzed in appropriate Shank deficient mouse models. Here we will focus on the functional analysis of missense mutations found in *SHANK* genes. The relevance of most of these mutations for Shank function, and their role in autism pathogenesis is unclear. This is partly due to the fact that mutations spare the most well studied functional domains of Shank3, such as the PDZ and SAM domains, or the short proline-rich motifs which are required for interactions with postsynaptic partners Homer, Cortactin, dynamin, IRSp53 and Abi-1. One set of mutations affects the N-terminal part, including the highly conserved SPN domain and ankyrin repeats. Functional analysis from several groups has indicated that these mutations (e.g., R12C; L68P; R300C, and Q321R) interfere with the critical role of Shank3 for synapse formation. More recently the structural analysis of the SPN-ARR module has begun to shed light on the molecular consequences of mutations in the SPN of Shank3. The SPN was identified as a Ras association domain, with high affinities for GTP-bound, active forms of Ras and Rap. The two autism related mutations in this part of the protein, R12C and L68P, both abolish Ras binding. Further work is directed at identifying the consequences of Ras binding to Shank proteins at postsynaptic sites.

## Autism spectrum disorders (ASD)

Autism spectrum disorders (ASD) are neurodevelopmental disorders characterized by delayed acquisition of speech, deficits in social interactions and stereotypic behaviors. In recent years several molecular genetic studies in large cohorts of patients have shown that the pathogenesis of ASDs involves a strong genetic component (Leblond et al., 2014). In several cases, potentially pathogenic mutations were identified in genes coding for synaptic proteins (Kelleher et al., 2012). These include cell adhesion proteins of the Neuroligin and Neurexin families (Jamain et al., 2003); proteins involved in signaling (e.g., regulators of small G-protein signaling such as Epac or SynGAP Woolfrey et al., 2009; Clement et al., 2012); and scaffold proteins of excitatory, glutamatergic synapses, including all three members of the Shank family (Durand et al., 2007; Moessner et al., 2007; Gauthier et al., 2009; Berkel et al., 2010). Based on these findings autism has been considered as a synaptic disease or synaptopathy, with individual mutations suspected to affect synapse formation and/or synaptic signal transduction and plasticity.

Since the introduction of exome sequencing into human genetic diagnostics, many more sequence variants continue to be discovered in ASD patients. The diagnostic challenge is then for a given patient to navigate through all these variants and make a more or less educated guess whether the variation at hand is benign or likely to be pathogenic and causative to disease. This is particularly true for missense mutations where it is difficult to assess pathogenicity in the absence of further knowledge of the functional characteristics of the encoded protein.

## Shank/ProSAP proteins

Shank/ProSAP proteins (Shank1–3) are major scaffold proteins of the postsynaptic density (PSD); via multiple interactions they connect different types of glutamate receptor complexes with signaling molecules and the actin cytoskeleton of the dendritic spine. The ability of Shank3 to multimerize via its C-terminal SAM domain has led to the suggestion that formation of Shank clusters is a key event in PSD assembly (Baron et al., 2006). Shank proteins appear to function entirely through the establishment of molecular interactions with other PSD proteins; for this they employ a number of protein interaction motifs which are depicted in Table 1.

**Table 1:** Functional effects of mutations affecting the N-terminus of Shank3, the upper graphic shows the domain structure of Shank3, and lists the missense mutations found in autistic patients which affect the N-terminal part of the protein.

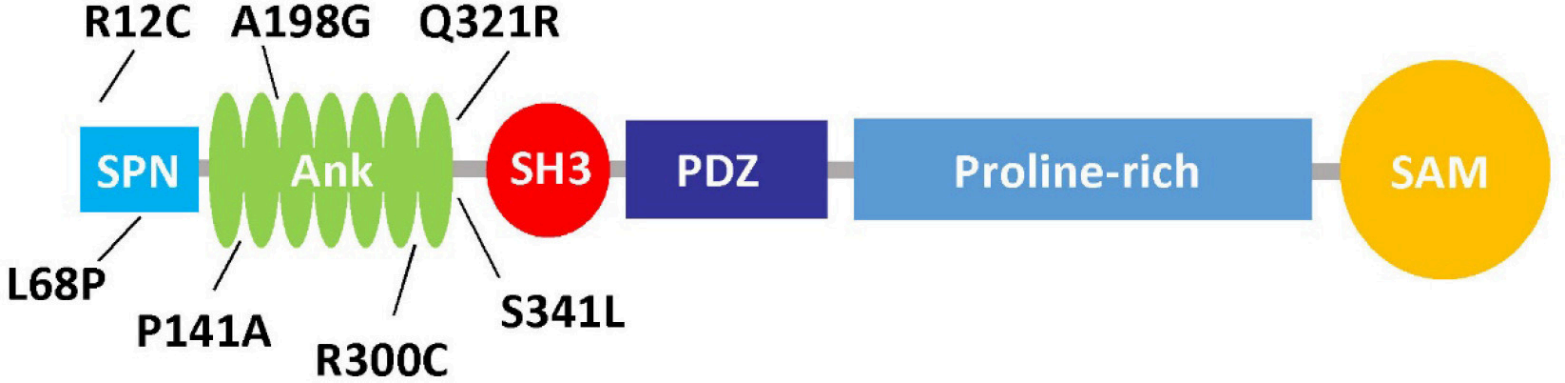						
**Alteration**	**References**	**Inheritance**	**Domain**	**Effect on mol. interactions**	**Effect on dendritic spines**	**Effect on synaptic signaling**
R12C	Durand et al., 2007	Mother (social phobia)	SPN	Ras: reduced Intramolecular: none	Density: moderate reduction Morphology: no effect F-actin: moderate reduction	Trans-synaptic: disrupted Support of mGluR5 pathway: disrupted Inhibition of integrin: lost
L68P	Gauthier et al., 2009	Father (epilepsy)	SPN	Ras: lost Fodrin: up (ns) Sharpin: up Intramolecular: lost	Density: no effect	Inhibition of integrin: lost
P141A	Boccuto et al., 2013	*de novo*	Ank	n.d.	n.d.	n.d.
A198G	Durand et al., 2007	Mother (healthy)	Ank	n.d.	n.d.	n.d.
R300C	Durand et al., 2007	Mother (healthy)	Ank	Fodrin: none Sharpin: up	Density: moderate reduction Morphology: no effect F-actin: moderate reduction	Trans-synaptic: disrupted
Q321R	Moessner et al., 2007	*de novo*	Ank	Fodrin: none Sharpin: up	Density: moderate reduction Morphology: shorter spines with larger heads F-actin: no effect	Trans-synaptic: disrupted
S341L	Moessner et al., 2007	Father (healthy)	Ank	n.d.	n.d.	n.d.

*N.d, not determined; ns, the observed effect was not statistically different from WT*.

Loss of one copy of the region on chromosome 22 which includes the *SHANK3* gene leads to 22q13 deletion/Phelan-McDermid syndrome, which is associated with severe intellectual disability (Bonaglia et al., 2006). More generally, *SHANK* gene dosage appears to be important as duplication of the *SHANK3* gene also leads to a neurological phenotype (Han et al., 2013). In autism and also schizophrenia cases, insertions, deletions, nonsense and splice site mutations have been observed on one *SHANK3* allele which lead to loss or truncation of the protein. By introducing some of these changes into the genome of mice, Zhou et al. (2016) observed that different mutations may affect brain function in very different ways, depending on cell type and developmental stage. In addition to these presumably loss of function variants, a number of missense mutations have been found in individual autism patients (Durand et al., 2007; Moessner et al., 2007; Gauthier et al., 2009). Interestingly, the relevance of most of these mutations for Shank3 function, and their role in autism pathogenesis is unclear. This is partly due to the genetics of the patients; thus some variations found in *SHANK3* in autism patients are inherited from healthy parents (e.g., R12C and R300C), ruling out a dominant effect on disease. Whereas, all other mutations are extremely rare, R300C is found 15 times in a database of exomes of 64.000, mostly healthy individuals (Exac database), suggesting that it could be a polymorphism rather than a pathogenic mutation. In contrast, P141A and Q312R variants occur *de novo* in the affected children (Moessner et al., 2007; Boccuto et al., 2013). The L68P mutant described by Gauthier et al. (2009) was inherited, but from an epileptic father, suggesting that it may affect neurological functions in different ways, dependent on environmental and other genetic factors.

A second problem lies in the fact that mutations spare the most well studied functional domains of Shank3. Thus, so far no mutations have been identified in patients which affect the PDZ and SAM domains, or those short sequence motifs which are required for interactions with postsynaptic partners Homer, Cortactin, dynamin, IRSp53, and Abi-1. Instead, missense mutations are found in the N-terminal region including the ankyrin repeats (see below), and in the long proline rich segment between PDZ and SAM domains which has been characterized as an “intrinsically disordered region.” As a consequence, it has been difficult to design functional assays where one could actually show that these mutations interfere with specific functions of the Shank3 protein. Intriguingly, mice lacking only the Ank repeat containing forms of Shank3 (Shank3A^−/−^ mice) show a much milder behavioral phenotype than mice lacking most variants of the protein [Shank3B^−/−^ mice (Peça et al., 2011)]. Thus, the N-terminal part of Shank3 is highly affected by mutations in human patients, but its relevance in mutant mice is unclear. This leaves us with the more general open question whether rare missense mutations found in patients are indeed pathogenic, or simply represent extremely rare polymorphisms.

## Structure of the N-terminal region of Shank3

**Figure 1:**
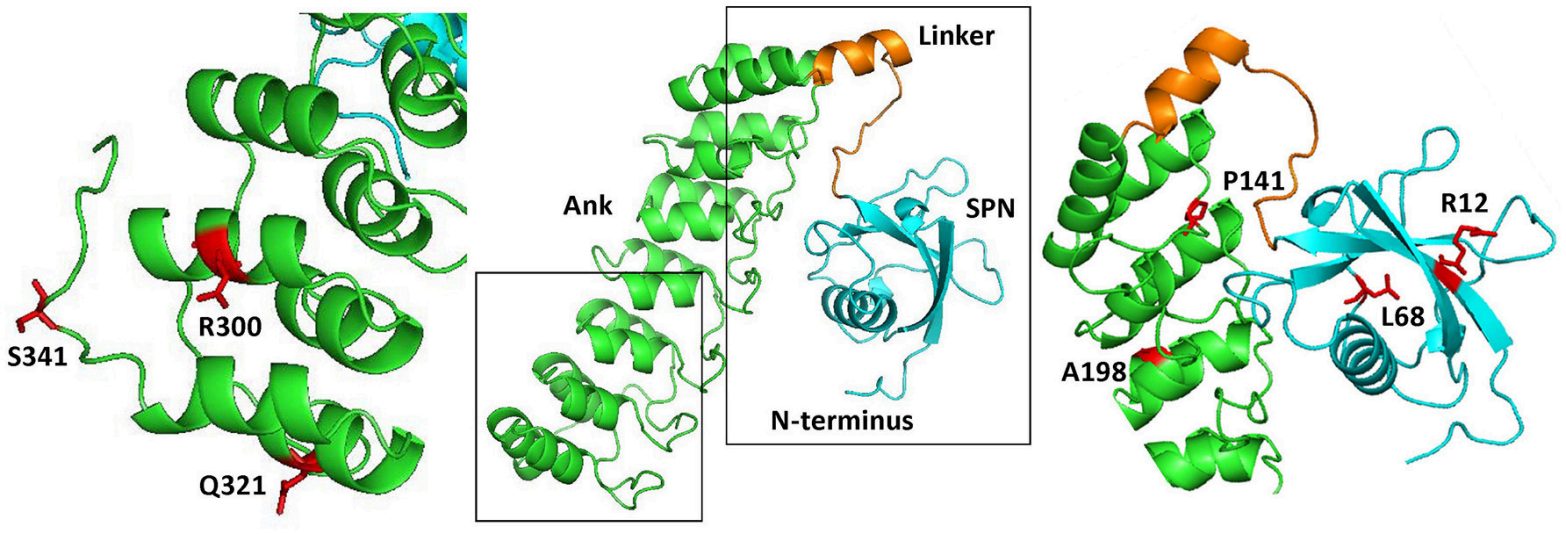
Three dimensional structure of the N-terminus of Shank3, comprising the SPN and Ankyrin repeat (Ank) domains (based on PDB entry 5G4X; Lilja et al., 2017). The central part shows an overview, where both the SPN domain and the set of seven Ankyrin repeats can be clearly seen as individual domains connected by a short linker. Both the linker region and SPN domain are engaged in an extensive intramolecular contact with the Ank domain. To the right and left, enlarged and slightly rotated pictures show in red the positions and side chains of residues which have been shown to be altered in autistic patients.

Long variants of Shank proteins contains in their N-terminal part a set of seven Ankyrin repeats, followed by the SH3 domain (the “Sh” and “ank” in Shank). In addition, the Ank domains are preceded by a conserved domain which was initially overlooked when Shank domains were assigned. We termed this the Shank/ProSAP N-terminal (SPN) domain. This about 90 amino acid domain is most similar to the so-called F_0_ motif in the FERM domain of talin, which itself is a major scaffold of focal adhesions (Goult et al., 2010). Using X-ray crystallography, Lilja et al. (2017) solved the three dimensional structure of an N-terminal fragment of rat Shank3, consisting of the SPN and Ank domains. Here the SPN domain was confirmed to fold in a ubiquitin like (ubl) fold, like the talin F_0_ domain. A set of seven ankyrin repeats was observed in this structure, which was linked to the SPN motif by a 19 amino acid long linker region. This linker and the SPN domain fold back against the Ank repeats, leading to an extended interface between both domains which confirms a previously detected intramolecular interaction between SPN and Ank (Mameza et al., 2013). The structure also provides a molecular framework for further analysis of missense mutations, as the positions of mutated residues can be viewed in a 3D model (Figure 1).

## Molecular interactions

Using both unbiased approaches such as yeast two hybrid screening, as well as educated guesses based on functional data, several interaction partners for the Ank domain have been identified: (a) Sharpin, a cytosolic signaling protein (Lim et al., 2001); (b) α-fodrin, a cytoskeletal protein which provides a link to the actin cytoskeleton (Bockers et al., 2001); (c) ion channels of the HCN family (Yi et al., 2016). Whereas, it is unclear whether Sharpin is present at postsynaptic sites in neurons together with Shank, both α-fodrin and HCN channels can be envisioned to play a role in the assumed function of Shank proteins, namely organizing the postsynaptic density and linking membrane proteins with the submembranous cytoskeleton. Intriguingly, the three interactors do not share any sequence homology or common sequence motifs which might predict a common way of binding to the Ank domain.

Initially we observed that the SPN domain is locked in an intramolecular interaction with the Ank domain, blocking access of Ank domain ligands α-fodrin and Sharpin (Mameza et al., 2013). The ubl fold of the SPN (and F_0_ of talin) is similar to that observed in Ras association domains. Indeed we observed that the SPN binds to active (GTP bound) Ras family members (HRas, KRas; as well as Rap1 variants) with high affinity, whereas GDP-bound Ras proteins do not bind.

## Molecular effects of missense mutations

The effects of several missense mutations found in autism patients on these interactions has been studied in some detail, mostly in biochemical assays using pulldown and co-immunoprecipitation experiments (see Table 1). Mutations in the Ank domain appeared to have a rather moderate effect on binding of this domain to either Sharpin or α-fodrin. In contrast, both mutations in the SPN domain (R12C and L68P) interfered with binding to Ras family small G-proteins. In the case of R12C, this could be rationalized by molecular modeling of the Shank3/Ras complex. Here, Arg12 is facing Ras and is involved in forming an ion pair with Glu37 of Ras. Mutation to Cysteine obviously eliminates this interaction and leads to loss of affinity for GTP-bound forms of HRas and Rap1. The L68P mutation interferes not only with Ras binding, but also with the intramolecular interaction with the Ank domain. Leu68 is located in the inner hydrophobic core of the SPN domain, and it is likely that a proline residue at this position will lead to an unfolding of the domain, thereby eliminating both interactions (with Ras and with the Ank domain). As a consequence, access to the Ank domain is no longer blocked by the SPN motif, leading to improved binding of Sharpin, α-fodrin and exogenously applied SPN domain (Mameza et al., 2013). These data suggest that the L68P mutation most likely induces a permanently open conformation of the N-terminal portion of Shank3, where the function of the Ank domain is unregulated and interaction with small G-proteins of Ras family eliminated (Mameza et al., 2013; Lilja et al., 2017).

## Functional effects of missense mutations in the formation of dendritic spines

Expression of missense mutant variants of Shank3 in primary hippocampal neurons showed that the normal synaptic targeting of Shank3 was not affected by these mutations (Arons et al., 2012; Durand et al., 2012). Overexpression of Shank3 WT significantly increased dendritic spine density and decreased the number of filopodia; Shank3 constructs carrying the N-terminal mutations showed a more moderate effect on spine formation (Arons et al., 2012; Durand et al., 2012). Shank3 WT affects spine maturation by forming spines with larger heads compared to control neurons transfected with GFP. However, Shank3 carrying the *de novo* mutation Q321R caused only a slight increase in the head width but also a decrease in the length of spines. Overexpression of Shank3 variants containing the two inherited mutations R12C and R300C had no significant effect on spine size; thus these mutations disrupt the role of Shank3 on spine maturation (Durand et al., 2012).

The reduction of mEPSC frequency in Shank3 knockdown neurons (expressing a shShank3 construct) was fully restored to control levels by transfection with a shShank3-resistant form of WT Shank3 and the L68P mutant, suggesting that the L68P mutant supports normal excitatory synaptic transmission and does in this respect not result in a loss of function (Mameza et al., 2013). In another study, overexpression of Shank3 WT in hippocampal neurons significantly increased mEPSC frequency compared to the control neurons transfected with GFP, while missense mutations (R12C, R300C, Q321R) increased mEPSC frequency but not as much as WT (Durand et al., 2012).

The ASD-associated mutations in the Ank domain affect F-actin content in dendritic spines. Overexpression of Shank3 WT increased the F-actin recruitment to the dendritic spines compare to control GFP transfected neurons. Here, R12C and R300C mutant constructs had an intermediate effect on the level of F-actin in the dendritic spines and the Q321R variant had no effect. Thus, these mutations disrupt the regulatory effect of Shank3 on actin dynamics and remodeling in spines and at the postsynaptic density. This effect of Shank3 is independent of the known Shank3 interaction partner Cortactin, as WT and control neurons did not show a significant difference in the postsynaptic levels of this important regulator of actin in dendritic spines (Durand et al., 2012).

## Functional effects of missense mutations in transsynaptic signaling

Shank3 has been reported to affect not only the postsynaptic but also presynaptic function of excitatory synapses, by regulating the activity of Neuroligin/Neurexin complexes. Though SAPAP is likely to be the most specific, high affinity interactor of Shank3 in the PSD (Zeng et al., 2016), the PDZ domain of Shank3 may also directly interact with the cytoplasmic tails of postsynaptic Neuroligins (Meyer et al., 2004). Using quantitative immunofluorescent microscopy, Arons et al. (2012) showed that ASD-associated mutations in Shank3 (R12C, R300C, Q321R) interfere with the role of Shank3 in transsynaptic signaling. Unlike the overexpressed Shank3 WT, these three mutants failed to increase the level of postsynaptic Homer1 and presynaptic VGLUT1. In addition, the size of the total recycling pool of synaptic vesicles at presynaptic sites contacting dendritic profiles of neurons expressing these mutant variants of Shank3 significantly decreased compared to neurons expressing Shank3 WT. Paired whole-cell recordings showed that with all mutations, the amplitude of both AMPAR and NMDAR EPSCs decreased, and the synaptic failure rate increased compared to cells overexpressing Shank3 WT (Arons et al., 2012).

In a follow-up study, Arons et al. (2016) addressed the role of one of the mutations found in ASD patients (R12C) in the regulation of Shank3 function by Zinc. Using FRAP (fluorescence recovery after photobleaching) assays they showed that increases in the zinc level induces stabilization of Shank3 (R12C similar to WT) and that depletion of zinc with the zinc-specific reagent TPEN increase the fraction of the both WT and R12C mutant in the immobile pool. These data suggest that zinc sensitive dynamics of Shank3 (which likely depends on an intact SAM domain; Baron et al., 2006) was not altered by this mutation. Nevertheless, the inability of the R12C variant of Shank3 to mimic the effect of Shank3 WT in enhancing presynaptic levels of VGLUT1 after zinc treatment confirmed that it is incapable of *trans*-synaptic signaling (Arons et al., 2016).

## Functional effects of missense mutations in synaptic signaling

A study in 2011 showed that the R12C mutation impairs the role of Shank3 in supporting the mGluR5 pathway. Using DHPG as an agonist of group I mGluRs, it was observed that in neurons transfected with a Shank3 knockdown construct (shShank3), the mGluRI signaling pathway leading to phosphorylation of ERK1/2 and CREB was impaired. Overexpression of shShank3-resistant Shank3 WT and also full-length mGluR5 were able to rescue DHPG-induced ERK1/2 activation, while the Shank3 R12C mutant was not able to do so (Verpelli et al., 2011).

The ability of the SPN domain to bind active, GTP-bound Ras proteins might affect signal transduction at synapses in different ways. Thus, Shank3 (and Shank1) could be downstream effectors of Ras signaling, in a so far unidentified signaling pathway. Alternatively, Shank3 could function to sequester active G-proteins, thus limiting their availability for other pathways. Indeed, such a scenario has been observed as both Shank1 and Shank3 were reported to negatively regulate integrin activation. This occurs due to sequestration of active Rap1 proteins via the Shank SPN domain. Both SPN domain mutations (L68P and R12C) that disrupt the binding of Shank to G-proteins also impair the inhibitory effect of Shank3 on integrin activation. Overexpression of Shank3 constructs in the rat hippocampal neurons showed that WT Shank3, but not the L68P mutant suppress formation of filopodia by inhibiting integrin activity. Also, overexpression of Shank3 WT in Shank3 KO cortical neurons plated on laminin inhibited β1-integrin activity in neuronal growth cones, while overexpressed L68P mutant failed to do so (Lilja et al., 2017).

## Future perspectives and open questions

Particularly the N-terminal portion of Shank3 is affected by missense mutations found in autism patients. Therefore, we and others have attempted to analyze the functional relevance of these mutations on a structural, molecular and cell biological level. For several mutations it is now clear that they do affect molecular interactions of the Shank3 protein; in particular, the R12C mutation rather selectively affects the binding of small G-proteins. In contrast, the L68P appears to destroy the folding of the SPN domain, thereby disrupting all of its interactions and also its regulatory effect on the Ank repeats. While the role of Arg12 and Leu68 maybe explained by the structural analysis of the Shank3 N-terminus, this is much less clear for the other five mutations which alter residues in the Ank domain. This is due to the fact that, so far, we do not know how Ank interaction partners (Sharpin, α-fodrin, HCN1) actually bind to the Ank repeats. There is no single motif in either interaction partner which has been mapped with some precision, and there is also no consensus between these three possible partners. Further analysis, accompanied by structural analysis of a possible Ank/ligand complex might help to elucidate the relevance of ASD-associated variants here.

A second open question is: how do the deficits in molecular interactions which have been identified for several variants, actually correlate with the cellular phenotype observed in neuronal cells? This is currently most unclear for the R12C mutant, which alters F-actin content as well as the form of dendritic spines, which interferes with mGluR5 signaling and which disrupts the transsynaptic strengthening of synaptic connections (see Table 1). The SPN domain and Arg12 are far removed in linear sequence from the PDZ domain (which binds to Neuroligin) and from the homer binding motif which links Shank3 to the Homer/mGluR5 complex. The same holds true for Arg300 and Gln321 in the Ank motif; nevertheless, mutations in both residues also interfere with spine formation induced by Shank3, and with transsynaptic signaling. These observations suggest that a possible link between the Shank3 N-terminus and the actin-based cytoskeleton needs to be studied further. This link could be provided by the actin-associated α-fodrin (which binds to the Ank repeats). However, binding to α-fodrin is only slightly affected by the missense mutations discussed here. An alternative view, which has been suggested by Arons et al. (2016) is a possible switch of Shank3 between open and closed conformations. In this way, the N-terminal (SPN and Ank) domains would interact with the C-terminal, proline rich motifs which harbor binding sites for actin regulators (cortactin; Abi-1; IRSp53) and the homer/mGluR5 complex. Through this (so far undefined) interaction, it might be explained how the N-terminus of Shank3 leads to dysregulation of the well-studied C-terminal interactions of this protein. Again, both biochemical and structural analyses will be extremely helpful to elucidate these conformational changes.

## Author contributions

All authors listed have made a substantial, direct and intellectual contribution to the work, and approved it for publication.

### Conflict of interest statement

The authors declare that the research was conducted in the absence of any commercial or financial relationships that could be construed as a potential conflict of interest.
